# Obituary: Dr. Thomas Moore

**Published:** 1882-04

**Authors:** 


### OBITUARY.

### Death of Dr. Thomas Moore.

On Saturday morning, March 25th, Dr. Thomas Moore, one of the most distinguished homœopathic practitioners of Pennsylvania, died suddenly while on a visit to a patient who resided near his own home, in Germantown. As he was about leaving, he remarked that he had still nineteen visits to make, and that he felt ill. He sat down, leaned his head upon his hand, and a moment after exclaimed, "Oh, my heart!" The patient called for her husband, and he came in, only to find the physician unconscious. Medical aid was speedily summoned, but Dr. Moore expired very shortly after his exclamation of distress.

Dr. Moore was about fifty-five years of age, and graduated from the University of Pennsylvania in 1848. He practiced the "old school" system of treatment for about nine years, and then turned his attention to Homœopathy. He was a professor in the Homœopathic Medical College of Pennsylvania for three years, occupying the Chair of Anatomy, and for one year that of Obstetrics. He was at one time one of the city physicians for the Northern Liberties. In 1860 he removed to Germantown, where he built up an extensive practice. The deceased was the author of several important medical papers, among the most notable being one on the diet of infants.

It is with profound sorrow that we announce the death of Dr. Moore; his place, both as man and physician, will be difficult to fill. Such homœopathic practitioners as he are now but seldom found. He was a true Hahnemannian, a ripe scholar, and thorough Christian gentleman.

A special meeting of the Homœopathic Medical Society of the County of Philadelphia, was held March 28th, to take appropriate action.

